# Leaf economics of evergreen and deciduous tree species along an elevational gradient in a subtropical mountain

**DOI:** 10.1093/aobpla/plv064

**Published:** 2015-06-06

**Authors:** Kundong Bai, Chengxin He, Xianchong Wan, Debing Jiang

**Affiliations:** 1 Guangxi Key Laboratory of Plant Conservation and Restoration Ecology in Karst Terrain, Guangxi Institute of Botany, Chinese Academy of Sciences, Guilin 541006, China; 2 Institute of New Forest Technology, Chinese Academy of Forestry, Beijing 100091, China; 3 Guangxi Mao'er Mountain National Nature Reserve, Guilin 541316, China

**Keywords:** Bimodal distribution, deciduous tree species, elevation, evergreen tree species, leaf economics spectrum, phylogenetic comparative methods, phylogenetic distance

## Abstract

The ecophysiological mechanisms underlying the pattern of bimodal elevational distribution of evergreen tree species remain incompletely understood. Here we used leaf economics spectrum (LES) theory to explain such patterns. We measured leaf economic traits and constructed an LES for the co-existing 19 evergreen and 15 deciduous species growing in evergreen broad-leaved forest at low elevation, beech-mixed forest at middle elevation and hemlock-mixed forest at high elevation in Mao'er Mountain, Guangxi, Southern China (25°50′N, 110°49′E). Leaf economic traits presented low but significant phylogenetic signal, suggesting trait similarity between closely related species. After considering the effects of phylogenetic history, deciduous species in general showed a more acquisitive leaf strategy with a higher ratio of leaf water to dry mass, higher leaf nitrogen and phosphorous contents, higher photosynthetic and respiratory rates and greater photosynthetic nitrogen-use efficiency. In contrast, evergreen species exhibited a more conservative leaf strategy with higher leaf mass per area, greater construction costs and longer leaf life span. With the elevation-induced decreases of temperature and soil fertility, both evergreen and deciduous species showed greater resource conservation, suggesting the increasing importance of environmental filtering to community assembly with increasing elevation. We found close inter-specific correlations between leaf economic traits, suggesting that there are strong genetic constraints limiting the independent evolution of LES traits. Phylogenetic signal increased with decreasing evolutionary rate across leaf economic traits, suggesting that genetic constraints are important for the process of trait evolution. We found a significantly positive relationship between primary axis species score (PASS) distance and phylogenetic distance across species pairs and an increasing average PASS distance between evergreen and deciduous species with increasing elevation, implying that the frequency of distantly related evergreen and deciduous pairs with wide spreading of leaf economic values increases with increasing elevation. Our findings thus suggest that elevation acts as an environmental filter to both select the locally adapted evergreen and deciduous species with sufficient phylogenetic variation and regulate their distribution along the elevational gradient based on their coordinated spreading of phylogenetic divergence and leaf economic variation.

## Abstract (precis)

We used leaf economics spectrum (LES) theory to explain the bimodal elevational distribution of evergreen tree species, which is one of the most puzzling biogeographic patterns in the world. Our results suggest that elevation acts as an environmental filter to both select the locally adapted evergreen and deciduous species with sufficient phylogenetic variation and distinct leaf economic strategies and regulate their distribution along the elevational gradient based on their coordinated spreading of phylogenetic divergence and leaf economic variation. If species are filtered from regional species pools, changing climate may affect both the species and leaf economic composition of plant communities.

## Introduction

The patterns in the dominance of evergreen vs. deciduous tree species have intrigued ecologists for centuries, but remain incompletely understood (Monk 1966; Kikuzawa 1991; Givnish 2002; van Ommen Kloeke *et al.* 2012). Evergreen species tend to dominate sites where climatic seasonality is not distinct or where resources are difficult to obtain while deciduous species appear to be favoured wherever annual variation in temperature or precipitation results in marked favourable vs. unfavourable periods for carbon gain (Chabot and Hicks 1982; Reich *et al.* 1992). At the global scale, the relative frequency of evergreen species has a bimodal latitudinal distribution pattern. Evergreen broad-leaved species dominate tropical and subtropical regions, whereas evergreen needle-leaved species tend to inhabit in boreal regions. In contrast, deciduous broad-leaved species characterize temperate forests at mid-latitudes (Reich *et al.* 1992; Givnish 2002).

Bringing together leaf trait data spanning 2548 species from a wide range of vegetation types in tropical, subtropical, temperate and boreal regions, Wright *et al.* (2004) developed the worldwide leaf economics spectrum (LES), which is running from a slow-return end encompassing species with high leaf mass per area (LMA), high leaf life span (LLS), low nitrogen (N_mass_) and phosphorous (P_mass_) contents and low leaf dry mass-based net photosynthetic rate (*A*_mass_) and respiration rate (*R*_mass_) to fast return end with the opposite suite of traits. Wright *et al*. (2005*a*) showed evidence that photosynthetic nitrogen-use efficiency (PNUE) can also be regarded as a component of LES, because PNUE tends to be higher for species at the quick-return end of LES. Shipley *et al.* (2006) suggested that LES is generated by two fundamental trades-offs: a necessary trade-off between allocation to structural tissues vs. liquid phase processes (e.g. leaf dry mass vs. leaf water mass) and an evolutionary trade-off between *A*_mass_, construction costs (CCs) and LLS. Freschet *et al.* (2010) provided evidence for the tight correspondence between LES and whole-plant economics spectrum in a subarctic flora, indicating the critical role of LES in determining the whole-plant ecological strategies. In short, these different leaf trait combinations represent alternative leaf economic strategies for balancing the cost of constructing a leaf vs. the carbon fixation return (i.e. rapid resource acquisition vs. great resource conservation) (Donovan *et al.* 2011).

Because leaf economic traits and trait relationships are modulated by climate and soil nutrient status, LES can reflect adaptation to both small- and large-scale environmental gradients. In general, species at hotter, drier and more infertile sites tend to be closer to the slow-return end of LES due to their relatively higher LMA and longer LLS (Wright *et al.* 2005*b*; Ordoñez *et al*. 2009; Freschet *et al.* 2010). It is noteworthy that the variation in LLS is best explained by temperature, instead of by combinations of temperature, water and nutrient availability (van Ommen Kloeke *et al.* 2012). As mean annual temperature (MAT) decreases, evergreen species extend LLS at the expense of high LMA while deciduous species decrease LLS at the compensation of high nutrient contents and *A*_mass_ (van Ommen Kloeke *et al.* 2012; Kikuzawa *et al.* 2013). Consequently, these findings together give us a clue to the understanding of the bimodal latitudinal distribution pattern of evergreen species from a cost–benefit perspective. In tropical and subtropical regions where unfavourable temperature seasons are relatively short, the retention of evergreen leaves is beneficial to carbon fixation. However, in temperate regions where unfavourable temperature seasons become longer, the maintenance of canopy leaves during unfavourable seasons requires the vast investments of nutrients and dry mass. If such investments of nutrients and dry mass are larger than the cost of new leaf construction, the deciduous strategy is favoured. As unfavourable seasons prolong further, it is difficult for deciduous species to fix enough carbon to compensate for the investments of nutrients and dry mass in the growing seasons. Therefore, longer LLS is selected to prolong the accumulation of carbon (Kikuzawa 1991, 1995; Givnish 2002; Wright *et al.* 2004; 2005*b*; Kikuzawa *et al.* 2013).

Although LES effectively segregates species according to the variation in leaf economic strategies in an environment or across environmental gradients, relatively little is known about its evolution. The strong and consistent cross-species correlations of LES traits require explicit consideration of evolutionary history, because for any phylogenetically linked taxa, phenotypic similarity may reflect convergent evolution or limited evolutionary distance from a shared common ancestor (e.g. an internal node in a phylogeny), or both (Felsenstein 1985; Harvey and Pagel 1991). Some studies have provided empirical evidence that highlights the role of phylogeny in the evolution of LES. For instance, most leaf economic traits at both local and global scales have been found to present phylogenetic signal, which may reflect that phylogenetic relatedness can serve as a proxy for trait similarity (Penuelas *et al.* 2010; Walls 2011). Moreover, a few cross-species comparative studies using phylogenetically independent contrasts (PICs) have shown clear evidence for the coordinated evolution between leaf economic traits such as between LLS and LMA (Ackerly and Reich 1999; Mediavilla *et al.* 2008). However, estimation of PICs and their statistical analysis require an assumption that continuous characters evolve by Brownian motion, which is a random walk with rates of evolutionary change per unit branch length constant in all branches of the phylogeny. The Brownian motion-type evolution means that LES traits present similar tempo of evolution during the long-term natural selection (Garland *et al.* 1992; Revell *et al.* 2008). Although researches that explore the evolution of continuous traits tend to support the Brownian motion model, it is widely acknowledged that trait differences in sympatric species can diversify rapidly, against the Brownian model (Ackerly 2009; Valente *et al.* 2010). Finally, evolutionary distance relates to functional distance along a single trait axis or within a multivariate space at species-pairwise level, which can result in more intense competition for resources between closely related than between more distantly related species (Cahill *et al.* 2008; Cadotte *et al.* 2013). Overall, it is very important to investigate whether the evolution of leaf economic traits is best-fitted by the Brownian motion model, detect the evolutionary rate of leaf economic traits and explore whether species-pairwise evolutionary distance is linked to economic distance along the LES.

In the subtropical montane zones of Southern China, the elevational distribution pattern of evergreen and deciduous species resembles the global latitudinal distribution pattern (Wu 1980; Cao 1995; Kikuzawa 1996). At low elevations, the frequency of evergreen species is high and, at middle elevations, the frequency of deciduous species becomes high. At even higher elevations, however, evergreen species dominate again. Similar to the latitude-induced change in MAT, MAT tends to decrease with increasing elevation (Körner 2007). This elevation-induced change in MAT can result in different leaf economic responses of evergreen and deciduous species to elevation. For example, evergreen species have been found to increase LLS and LMA but decrease *A*_mass_, N_mass_ and PNUE with the elevation-induced decrease of MAT, suggestive of the greater recourse conservation in lower temperatures at higher elevations (Kikuzawa and Kudo 1995; Cordell *et al.* 1998; Scheepens *et al.* 2010). Conversely, deciduous species tend to decrease LLS but maintain a positive carbon balance at high elevations by having leaves with low LMA and high N_mass_, indicating a greater resource acquisition in lower temperatures at higher elevations (Kikuzawa and Kudo 1995; Takahashi and Miyajima 2008).

Here we applied the LES theory to explain the bimodal elevational distribution pattern of evergreen tree species, which received little attention in previous researches. We examined the leaf economic traits and constructed an LES in co-existing evergreen and deciduous species at different elevations in Mao'er Mountain, Guangxi, Southern China. Different forest types along the elevational gradient exist: evergreen broad-leaved forest (<1300 m), beech-mixed forest (1300–1800 m) and hemlock-mixed forest (>1800 m) (Cao 1995; Huang and Jiang 2002; Zhu *et al.* 2004). We argue that LES would effectively separate evergreen and deciduous species and reflect adaptation to the elevational gradient in Mao'er Mountain. Specifically, we had the following predictions: (i) at each elevation, deciduous species show acquisitive leaf economics while evergreen species have conservative leaf economics. (ii) With the increase in elevation, evergreen species become more conservative while deciduous species are more acquisitive. (iii) Leaf economic shifts between leaf habits and among elevations are related to phylogenetic distance.

## Methods

### Study site and plant materials

**Figure 1.**
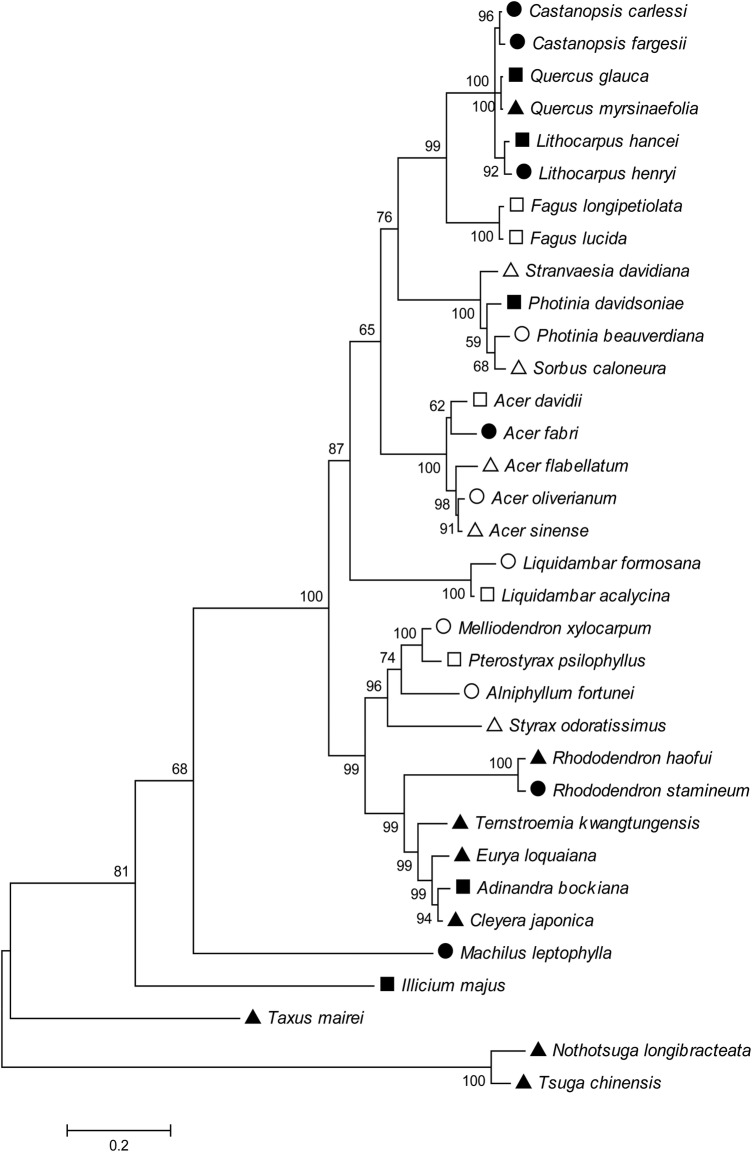
Phylogenetic tree constructed by the maximum likelihood (ML) method using the nuclear internal transcribed spacer (ITS) sequences of the 19 evergreen (filled symbols) and 15 deciduous species (open symbols) at low (circles), middle (squares) and high (triangles) elevations in Mao'er Mountain. The bootstrap values are shown at branching points.

This study was conducted in three typical forest types (i.e. evergreen broad-leaved forest with montane subtropical climate at low elevation, beech-mixed forest with montane warm temperate climate at middle elevation and hemlock-mixed forest with montane moderate temperate climate at high elevation) in Mao'er Mountain (25°50′N, 110°49′E.), Guangxi, Southern China (Cao 1995; Huang and Jiang 2002; Zhu *et al.* 2004). The mountain, with its highest peak of 2142.5 m above sea level (a.s.l.), is part of Nanling Mountain Range that partially blocks the cold winds coming from northern China in the winter. According to the weather station at 1200 m a.s.l., MAT and mean annual precipitation were 12.8 °C and 2509 mm, respectively. Mean summer temperature through June to August was 20.8 °C. We selected 34 tree species from 11 families in the three forest types. Most species were dominant or subdominant species. Specially, eight species from the Fagaceae family were typical constructive species in montane zones in Southern China (Fig. 1; **Supporting Information—Table S1**; Huang and Jiang 2002).

### Measurements of leaf and environmental traits

Leaf traits were measured on 5–8 mature individuals per species in three plots at 900, 1500 and 1900 m a.s.l. from 2008 to 2012. The size of each plot was around 8000 m^2^ (i.e. 80 × 100 m). Here we defined an individual as a mature plant as long as it was found to bloom and bear seeds, so the average height of sampled individuals was around 7 m. Since leaf traits (e.g. LMA and photosynthesis) could change with leaf age, and LLS also varied widely among species, parameters were measured on the fully expanded young leaves in all species to standardize physiological leaf age. Sun leaves were selected on plants growing in relatively open situations for all species to minimize the effects of light environment.

In order to estimate LLS of the 31 broad-leaved species, we monitored leaf emergence and senescence using tags and drawings for 15 leaves in each individual plant. The census intervals ranged from every week during the peak leaf production time between March and May and the senescence period in October–December to every month during the other growing seasons. We calculated LLS as the duration between leaf emergence and senescence for each individual leaf and took the average per individual plant and per species (Reich *et al.* 1991). For the three needle-leaved species, we selected 10 branches in each individual plant and estimated the average LLS by counting the number of annual cohorts with at least 50 % of their leaves retained on each branch (Reich *et al.* 1999). Although this method could give a slight overestimate of LLS because of some mortality in younger cohorts, it is still considered as an easy and effective way to estimate LLS for needle-leaved species (Pérez-Hanguindeguy *et al.* 2013).

On sunny days in summer, gas exchange measurements were conducted. These measurements were performed in the field between 8:00 and 11:30 solar time with an Li-6400 portable photosynthesis system (Li-Cor, Lincoln, NE, USA). Maximal photosynthetic rate (*A*) and dark respiration rate (*R*) were measured at 1500 and 0 μmol m^−2^ s^−1^ photosynthetic photon flux density with a stable atmospheric CO_2_ concentration of 380 μmol mol^−1^, respectively. Leaf temperature was controlled at 20 °C and leaf-to-air vapour pressure deficit was <1.0 kPa. Gas exchange was not measured until the leaf was fully induced at each light level, which was determined visually using the graphic interphase in the Li-6400. Because of the small size of leaves of the needle-leaved species, gas exchange was measured on several leaves enclosed simultaneously and manoeuvered to occupy most or the entire chamber window, without overlapping. In cases where leaves did not occupy the entire window, the gas exchange was calculated with the actual leaf area of the enclosed sample. We took a total of 15–25 measurements per species from different individuals and averaged these for subsequent analyses.

After gas exchange measurements, leaves were harvested and immediately sealed in plastic bags. After leaf fresh weight was measured, leaf area was measured with a leaf area meter (Li-3000A; Li-Cor). Then, the leaves were oven-dried for 48 h at 65 °C, for determining LMA (dry mass/area), the ratio of leaf water to dry mass (*W*_m_), carbon content (C) using the Walkley–Black wet oxidation method, N content by the micro-Kjeldahl method and P content using atomic absorption spectrum-photometry. Photosynthetic nitrogen-use efficiency was calculated as mass-based *A*/N. The CC of leaf tissue (grams of glucose necessary to synthesize 1 g leaf tissue) was calculated as (5.39 × C − 1191)/1000 (Vertregt and Penning de Vries 1987).

In each plot, soil samples were randomly collected for the upper 20 cm of soil with five replicates in August 2012. Air-dried, root-removed and ground samples were passed through a 100-mesh sieve. We used some of the most common measures (i.e. soil total N, soil total P, soil N/P and soil C/N) to evaluate soil fertility (Cleveland and Liptzin 2007; Ordoñez *et al.* 2009). We analysed the contents of C, N and P in soils using the same ways in leaves. The MAT in each plot was estimated through the increase of 0.55 °C per 100 m decrease of elevation (Huang and Jiang 2002), using the climatic database in the weather station at 1200 m a.s.l. from 2008 to 2012.

### Construction of the phylogenetic tree

The phylogenetic relationships among the 34 species were inferred from ITS (internal transcribed spacer 1, 5.8S ribosomal RNA, internal transcribed spacer 2) sequences. Internal transcribed spacer sequences were retrieved from GenBank **[see Supporting Information—Table S1]**. Alignment of ITS sequences was performed using ClustalW. We used hierarchical Bayesian information criterion (Schwarz 1978) test to select the best model of nucleotide substitution, which was a Tamura–Nei (TN; Tamura and Nei 1993) model, allowing for rate heterogeneity across sites assuming a discrete Gamma distribution and for a proportion of sites to be invariable (TN + G + I). The phylogenetic tree was then inferred by the maximum likelihood (ML) method based on the TN model. The trees were evaluated using the bootstrap test based on 1000 replicates. The tree with the highest log-likelihood (−5721.6) was shown. The tree was drawn to scale, with branch lengths measured in the number of substitutions per site (Fig. 1). Pairwise phylogenetic distance between species was also conducted based on the TN model. All the evolutionary analyses were conducted using MEGA5.2 (Tamura *et al.* 2011; Hall 2013).

### Statistics and phylogenetic comparative methods

Difference in MAT, soil total N, soil total P, soil C/N or soil N/P among elevations was tested with one-way analysis of variance after data log_10_-transformation. All species mean values of leaf economic traits were also log_10_-transformed prior to analysis to increase the normality of distribution (Kerkhoff and Enquist 2009; **Supporting Information—Table S1**). Principal component analyses (PCAs) were performed using nine leaf traits (Revell 2009). Because of the high percentage of variance explained by the primary PCA axis, the primary axis species score (PASS) was used in the subsequent analyses as a proxy for leaf economics. Leaf economic distance along the primary axis, which was calculated as the absolute value of Species A score minus Species B score, could serve as a proxy for niche distance and reflect the competitive intensity between pairwise species (Cadotte *et al.* 2013). In order to illustrate the influence of phylogeny on leaf economics, we firstly used linear regression analysis to test the relationship between PASS distance and phylogenetic distance across all species pairs. However, inter-elevational pairs could rarely co-occur in the same natural environment (e.g. pair of *Nothotsuga longibracteata* at high elevation vs. *Castanopsis fargesii* at low elevation; Fig. 1) and the comparison of evergreen and deciduous species should be considered with their phylogenetic relatedness (Antúnez *et al.* 2001). We then conducted linear regression analysis to test for the relationship between PASS distance and phylogenetic distance using all intra-elevational pairs of evergreen vs. deciduous species. These above analyses were conducted in SPSS 13.0 (SPSS, Chicago, IL, USA).

To perform the comparisons across leaf traits, we employed the Pagel's λ, which is a quantitative measure of phylogenetic dependence introduced by Pagel and varies continuously from zero to unity (Pagel 1999). We tested if λ was significantly different from zero (i.e. no phylogenetic signal) or unity (i.e. the Brownian expectation) using likelihood ratio tests comparing a model with the observed ML value of λ to a model with a fixed λ of zero or unity. If λ was found to be the intermediate values between zero and unity, it indicated phylogenetic signal in the trait that had evolved according to a process other than pure Brownian motion (Kamilar and Cooper 2013). Because we found that the leaf traits presented low but significant phylogenetic signal **[see Supporting Information—Table S2]**, phylogenetic correlation was necessary in subsequent regression analyses. We used phylogenetic generalized least square (PGLS) regressions for regression analyses. Phylogenetic generalized least square controls for phylogenetic relatedness by adjusting the expected variance and co-variance of regression residues employing the matrix of phylogenetic distance, which is mathematically similar to analysing the data employing PICs but can depart from a strict Brownian motion process (Orme *et al*. 2012). First, we employed PGLS to analyse how leaf economic traits and PASS varied as a function of leaf habit or elevation that was included as an independent categorical variable. Second, we explored the inter-specific corrections between leaf economic traits using PGLS. Finally, we used PGLS to determine how the PASS of evergreen and deciduous species varied as function of MAT, soil C/N and soil N/P.

We compared the basic Brownian motion and Ornstein–Uhlenbeck (OU) models of trait evolution in an attempt to explore the relationships among phylogenetic signal, evolutionary process and rate (Butler and King 2004; Gonzalez-Voyer and Kolm 2011), based on the finding that the evolution of LMA is well-suited to the OU model at clade level in a large dataset of vascular plants (Flores *et al.* 2014). The OU model is a very simple evolutionary model incorporating selection and different from the Brownian model in that it possesses a selective optimum, given by the value of α (Butler and King 2004). Under the OU model, the rate of trait change along the branches of a phylogeny depends on either the distance between the actual trait value and the value of the selective optimum or the strength of the ‘pull’ towards the selective optimum. The rate of trait evolution along the branches of the phylogenetic tree will be increasingly faster with the increase of α, as compared with the basic Brownian process (Butler and King 2004; Gonzalez-Voyer and Kolm 2011). For each trait, we compared the fit of the Brownian motion model with the fit of the OU model using a log-likelihood ratio test. We used the comparison between the two evolutionary models and estimate of α as a measure of the strength of selection acting on traits. We fitted the OU model with a single optimum, instead of more complex models with multiple optimum because we had no *a priori* independent means of estimating potentially different selection regimes for each trait (Butler and King 2004; Gonzalez-Voyer and Kolm 2011). The value of α was then regarded as an estimate of the tempo of trait evolution. In order to explore the relationship between evolutionary rate and phylogenetic signal, we performed Pearson's correlation analysis between α and λ across nine traits. All the above analyses in a phylogenetic context were conducted in the packages of GEIGER, PHYTOOLS and CAPER in R version 3.1.0 (Harmon *et al.* 2008; R Development Core Team 2008; Orme *et al.* 2012; Revell 2012).

## Results

### Phylogenetic signal and evolutionary rate

**Figure 2.**
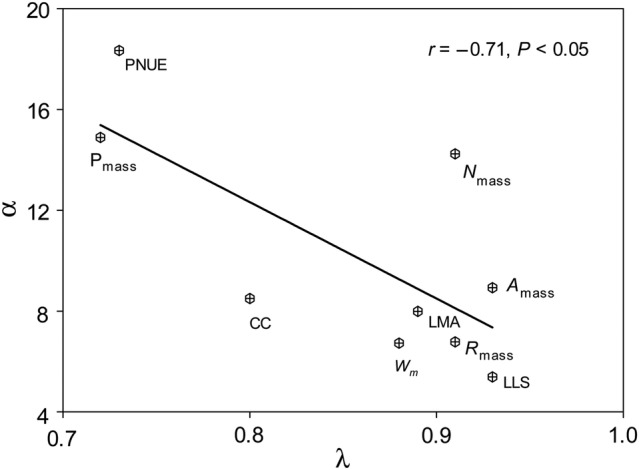
Relationship between phylogenetic signal (λ) and evolutionary rate (α) across nine leaf economic traits in Mao'er Mountain. Trait abbreviations are as defined in Table 1.

The nine leaf economic traits had λ values ranging from 0.72 (P_mass_) to 0.93 (LLS and A_mass_) **[see Supporting Information—Table S2]**. All the λ values were significantly higher than 0 but significantly lower than 1 **[see Supporting Information—Table S2]**, indicating low but significant phylogenetic signal. The OU model explained the evolution of leaf economic traits better than the Brownian model, as evidenced by higher log-likelihood values in the former (Table 1). The α values ranged from 5.39 (LLS) to 18.34 (PNUE); moreover, the α values of N_mass_, P_mass_ and PNUE tended to be higher than other leaf economic traits (Table 1). Across nine traits, α was significantly and negatively correlated with λ (Fig. 2).

**Table 1.** Estimation of the tempo of evolution of leaf economic traits. Log-likelihood values of the Brownian motion and Ornstein–Uhlenbeck (OU) models are shown. The α parameter of the OU model describes the strength of selection acting on a trait; higher values indicate faster evolution. The two models of evolution are compared via a log-likelihood ratio test and the table presents the *P*-value of the test. LLS, leaf life span (months); LMA, leaf mass per unit area (g m^−2^); CC, construction cost (g glucose g^−1^); *W*_m_, the ratio of leaf water to leaf dry mass; N_mass_, nitrogen content per dry mass (mg g^−1^); P_mass_, phosphorus content per dry mass (mg g^−1^); *A*_mass_, maximal photosynthetic rate per dry mass (nmol g^−1^ s^−1^); *R*_mass_, dark respiration rate per dry mass (nmol g^−1^ s^−1^); PNUE, photosynthetic nitrogen-use efficiency (μmol mol^−1^ s^−1^); PASS, primary axis species score.

	Brownian motion		Ornstein–Uhlenbeck model	
	Log-likelihood	α	Log-likelihood	*P*-value
LLS	−2.68	5.39	1.91	0.002
LMA	9.92	7.99	16.09	<0.001
CC	37.51	8.50	43.54	<0.001
*W*_m_	25.39	6.73	30.45	0.001
N_mass_	29.05	14.24	38.39	<0.001
P_mass_	14.36	14.89	22.53	<0.001
*A*_mass_	2.85	8.93	9.42	<0.001
*R*_mass_	7.64	6.78	12.83	0.001
PNUE	5.16	18.34	15.26	<0.001

### Effects of leaf habit and elevation on leaf economic traits

After considering the effects of phylogenetic history, evergreen species had significantly higher LLS, LMA and CC than deciduous species which in turn exhibited significantly greater *W*_m_, N_mass_, P_mass_, *A*_mass_, *R*_mass_ and PNUE at each elevation (Table 2). With the increase of elevation, LMA and CC increased significantly in both evergreen and deciduous species, but *W*_m_, *A*_mass_, *R*_mass_, P_mass_ and PNUE decreased significantly in both groups (Table 2). Leaf life span was greatest in evergreen species but lowest in deciduous species at high elevation (Table 2). Nitrogen content per dry mass was lowest in evergreen species at high elevation but did not vary systematically with elevation in deciduous species (Table 2).

**Table 2.** Leaf economic traits and PASS in evergreen and deciduous species at different elevations in Mao'er Mountain. Means ± standard deviations and *P*-values of the PGLSs regression using leaf habit or elevation as an independent categorical variable are shown. Abbreviations are as defined in Table 1.

Trait	Leaf habit	Low elevation	Middle elevation	High elevation	*P*-value
LLS	Evergreen	17.0 ± 3.3	21.1 ± 4.5	30.9 ± 9.1	<0.001
	Deciduous	8.3 ± 1.6	6.9 ± 1.5	5.3 ± 0.8	0.001
	*P*-value	<0.001	<0.001	<0.001	
LMA	Evergreen	123 ± 15	149 ± 18	183 ± 34	<0.001
	Deciduous	66 ± 14	69 ± 16	94 ± 19	0.004
	*P*-value	<0.001	<0.001	<0.001	
CC	Evergreen	1.66 ± 0.13	1.84 ± 0.15	2.02 ± 0.27	0.003
	Deciduous	1.29 ± 0.12	1.44 ± 0.13	1.62 ± 0.14	<0.001
	*P*-value	<0.001	<0.001	0.037	
*W*_m_	Evergreen	1.16 ± 0.12	1.07 ± 0.12	0.77 ± 0.13	<0.001
	Deciduous	1.55 ± 0.09	1.47 ± 0.10	1.21 ± 0.09	<0.001
	*P*-value	<0.001	<0.001	<0.001	
N_mass_	Evergreen	20.9 ± 2.3	21.1 ± 1.9	16.9 ± 1.8	<0.001
	Deciduous	25.6 ± 1.8	25.3 ± 1.0	28.0 ± 5.1	0.480
	*P*-value	0.002	0.001	<0.001	
P_mass_	Evergreen	1.80 ± 0.15	1.62 ± 0.19	0.97 ± 0.19	<0.001
	Deciduous	2.16 ± 0.22	1.84 ± 0.08	1.48 ± 0.10	<0.001
	*P*-value	0.004	0.026	<0.001	
*A*_mass_	Evergreen	92 ± 13	80 ± 13	52 ± 12	<0.001
	Deciduous	183 ± 25	175 ± 46	127 ± 32	0.002
	*P*-value	<0.001	<0.001	<0.001	
*R*_mass_	Evergreen	8.5 ± 1.9	7.9 ± 1.5	4.2 ± 1.2	<0.001
	Deciduous	13.6 ± 2.1	12.6 ± 2.0	9.8 ± 2.2	0.002
	*P*-value	0.001	<0.001	<0.001	
PNUE	Evergreen	62 ± 12	52 ± 6	42 ± 12	0.001
	Deciduous	101 ± 7	96 ± 25	64 ± 10	<0.001
	*P*-value	<0.001	<0.001	0.007	
PASS	Evergreen	−0.044 ± 0.271	−0.383 ± 0.326	−1.398 ± 0.439	<0.001
	Deciduous	1.201 ± 0.266	0.998 ± 0.303	0.474 ± 0.359	<0.001
	*P*-value	<0.001	<0.001	<0.001	

**Figure 3.**
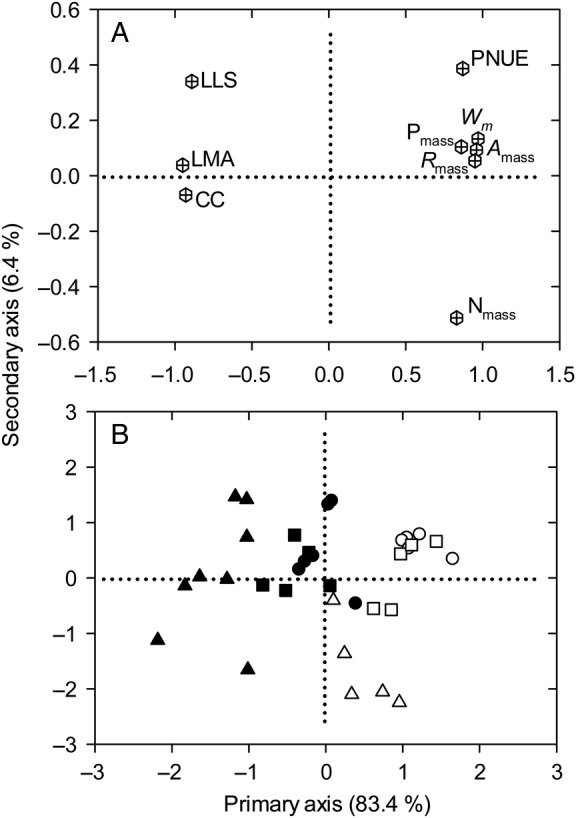
Principal component analysis for (A) the nine leaf traits and (B) the 19 evergreen (closed symbols) and 15 deciduous species (open symbols) at low (circles), middle (squares) and high (triangles) elevations in Mao'er Mountain. Trait abbreviations are as defined in Table 1.

The primary PCA axis explained 83.4 % of variation in the all species trait means dataset, as against only 6.4 % for the secondary axis (Fig. 3). The same directionality of trait loadings and similarly high percentage of variance explained by the primary axis were also found in species at a given elevation **[see Supporting Information—Table S3]**. Leaf life span, LMA and CC were negatively correlated with this primary axis of variation while the other traits were positively correlated with it. The primary axis thus indicated the alternative leaf economic strategies for higher resource acquisition vs. greater resource conservation. Greater resource acquisition was associated with greater *W*_m_, N_mass_, P_mass_, *A*_mass_, *R*_mass_ and PNUE, while higher resource conservation was associated with higher LLS, LMA and CC.

Primary axis species score was significantly affected by leaf habit at each elevation and differed significantly among elevations in both evergreen and deciduous species (Table 2), suggesting that species with different habits and from different elevations were well separated along the primary axis. Moreover, the average PASS distance between evergreen and deciduous species increased from 1.245 at low elevation to 1.874 at high elevation (Table 2).

### Correlations between leaf economic traits

Leaf economic traits tended to be closely inter-correlated after considering the effects of phylogenetic history (Table 3). For instance, *W*_m_, N_mass_, P_mass_, *A*_mass_ and *R*_mass_ were both negatively correlated with LMA, and CC was positively correlated with LMA. Moreover, LLS was correlated with *A*_mass_, *R*_mass_, N_mass_ and PNUE, reflecting its impact on the ability to acquire, use and conserve resources in evolution.

**Table 3.** Correlations between leaf economic traits across all tree species using PGLSs regression. Abbreviations are as defined in Table 1. Data were log_10_-transformed before analyses. Traits that are significantly correlated are marked: ***P < 0.001, **P < 0.01, *P < 0.05.

	LLS	LMA	CC	*W*_m_	N_mass_	P_mass_	*A*_mass_	*R*_mass_	PNUE
LLS									
LMA	0.86***								
CC	0.79***	0.93***							
*W*_m_	−0.82***	−0.83***	−0.89***						
N_mass_	−0.80***	−0.77***	−0.70***	0.73***					
P_mass_	−0.22	−0.64***	−0.83***	0.76***	0.54***				
*A*_mass_	−0.83***	−0.90***	−0.86***	0.90***	0.77***	0.74***			
*R*_mass_	−0.80***	−0.87***	−0.85***	0.93**	0.71***	0.85***	0.90***		
PNUE	−0.36*	−0.81***	−0.79***	0.79***	0.14	0.53***	0.91***	0.72***	

### Relationship between PASS and environmental and phylogenetic traits

**Figure 4.**
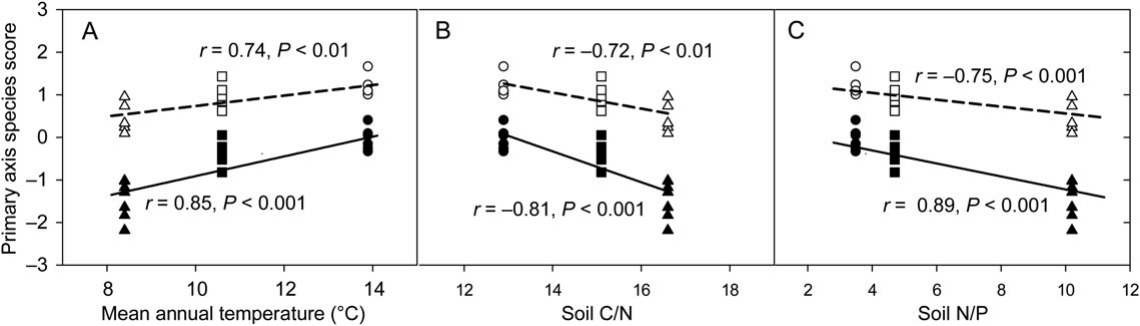
Relationships between PASS and (A) MAT, (B) the ratio of soil carbon content to nitrogen content (soil C/N) and (C) the ratio of soil nitrogen content to phosphorus content (soil N/P) in evergreen (filled symbols, solid lines) and deciduous species (open symbols, dashed lines) at low (circles), middle (squares) and high (triangles) elevations in Mao'er Mountain.

**Figure 5.**
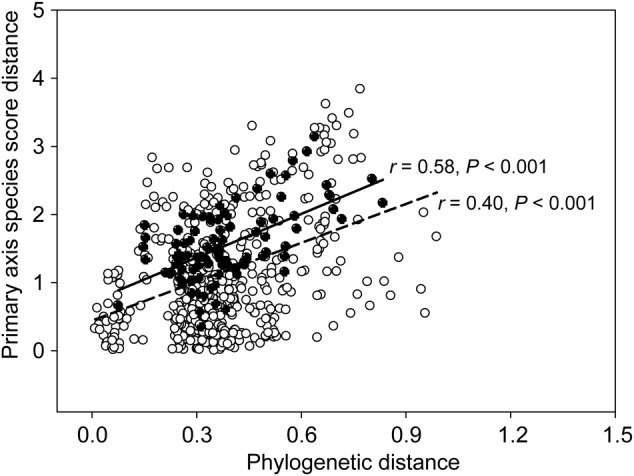
Relationship between PASS distance and phylogenetic distance across all species pairs (open symbols, dashed line) or intra-elevational pairs of evergreen vs. deciduous species (filled symbols, solid line) in Mao'er Mountain.

Mean annual temperature decreased significantly with increasing elevation (Table 4). Soil total N increased significantly with increasing elevation while soil total P was not significantly affected by elevation (Table 4). Both soil C/N and soil N/P increased significantly with increasing elevation (Table 4). Environmental variables representing the quality of soil organic matter (soil C/N), the status of soil nutrient limitation (soil N/P) and climate (MAT) were strong predictors of PASS (Fig. 4). The colder the environment was (i.e. lower MAT at high elevation), the more negative PASS was, i.e. the more resource conservative the species strategy was. Primary axis species score was found to decrease with increasing soil C/N and soil N/P. A significantly positive relationship between PASS distance and phylogenetic distance across all species pairs (*r* = 0.40, *P* < 0.001) or across the intra-elevational pairs of evergreen vs. deciduous species (*r* = 0.58, *P* < 0.001) was also found (Fig. 5).

**Table 4.** Soil and climatic traits at different elevations in Mao'er Mountain. Means ± standard deviations and *P*-values of the analysis of variance using elevation as a factor are shown.

Trait	Low elevation	Middle elevation	High elevation	*P*-value
MAT	13.9 ± 0.3	10.6 ± 0.3	8.4 ± 0.3	<0.001
Soil total N	0.38 ± 0.05	0.57 ± 0.07	0.89 ± 0.12	<0.001
Soil total P	0.118 ± 0.029	0.126 ± 0.026	0.089 ± 0.021	0.087
Soil C/N	12.9 ± 1.3	15.1 ± 2.4	16.6 ± 1.7	<0.05
Soil N/P	3.5 ± 1.1	4.7 ± 1.1	10.2 ± 1.3	<0.001

## Discussion

### Divergent effect of leaf habit on leaf economics

We found that evergreen species had higher LMA and CC, lower *W*_m_, N_mass_, P_mass_, *A*_mass_, *R*_mass_ and PNUE and longer LLS than deciduous species at each elevation (Table 2). Similar results have been reported in evergreen and deciduous species at local, regional and global scale (e.g. Reich *et al.* 1999; Wright *et al.* 2005*a*; Fu *et al*. 2012). Our result therefore confirms that the deciduous habit can be considered as an acquisitive leaf strategy while the evergreen habit is a conservative leaf strategy, consistent with our Prediction (i). The different strategies of leaf habits have different advantages in coping with the environmental conditions. Deciduous species are able to achieve higher carbon gain and ensuing higher growth at a lower leaf dry mass cost, thereby conferring a competitive advantage over evergreen species, especially when being co-existing with evergreen species in an environment where resources are not strongly limited (Reich *et al.* 1992; van Ommen Kloeke *et al.* 2012). In contrast, evergreen species have longer LLS at a higher leaf dry mass cost. Longer LLS increases the mean residence time of nutrients in the plant, which improves the overall nutrient-use efficiency and extends the photosynthetic season. However, leaf photosynthetic capacity in evergreen species has been found to be relatively lower (e.g. Reich *et al*. 1991; Wright *et al.* 2005*b*). Therefore, the main advantage of longer LLS in evergreen species resides in the higher nutrient retention potential in the plant, which enables them to be highly competitive and dominate in infertile habitats where natural selection favours traits such as longer LLS for their positive role in nutrient conservation (Pornon *et al.* 2011).

### Convergent effect of elevation on leaf economics

We found that PASS decreased with increasing elevation in both evergreen and deciduous species (Table 2), indicating that both evergreen and deciduous species in general became more conservative with the increase of elevation. Thus, this finding is contrary to our Prediction (ii). The convergent effect of elevation on leaf economics could largely result from the fact that both evergreen and deciduous species tended to increase LMA and CC and decrease *W*_m_, *A*_mass_, *R*_mass_, P_mass_ and PNUE with increasing elevation. Moreover, the increase of LLS and decrease of N_mass_ with increasing elevation also contributed to the elevation-induced change of resource conservation in evergreen species (Table 2). Note that the increase of resource conservation with increasing elevation in deciduous species in our study is contrary to the findings of Kikuzawa and Kudo (1995) and Takahashi and Miyajima (2008). The difference between our results and those obtained by them may be due to the fact that their measures of leaf traits were confined to a specific forest type where the co-existing species are under the same influence of climate but the length of favourable seasons could decrease with increasing elevation. In a specific forest type, the plastic responses of LLS in deciduous species to the elevation-induced varying length of favourable seasons could be adaptive for maximizing photosynthetic carbon gain (Kikuzawa and Kudo 1995). However, the forest type, climate and soil resource availability along the elevational gradient in our study were complicated. The tendency of increasing resource conservation with increasing elevation in both evergreen and deciduous species in our study lends support to the argument that selection imposed by elevation on linked traits results in trait convergence along similar elevational gradients (Read *et al.* 2014). Of course, tree species did not respond to elevation directly but rather to a suite of factors such as temperature and soil resource availability that covary with elevation.

Evergreen and deciduous species can compete intensively for soil resources, particularly in infertile habitats. Water availability is not likely to be a limiting factor for evergreen and deciduous species in our study mountain due to the sufficient level of precipitation (mean annual precipitation over 2500 mm; Huang and Jiang 2002). We found an increase of soil total N with increasing elevation and relatively stable soil total P across elevations (Table 4). However, soil nutrient contents are only very rough estimates of nutrient supply to the vegetation, because most of the soil nutrient stocks can be immobilized in the organic matter and consequently may be unavailable for most plants (Aerts and Chapin 2000; Ordoñez *et al.* 2009). We found soil C/N increased with increasing elevation (Table 4), suggesting the decrease of the quality of organic matter with increasing elevation (Ordoñez *et al.* 2009). The value of soil N/P at low, middle and high elevation was 3.5, 4.7 and 10.2, respectively (Table 4). All the ratios were very different from the global soil N/P breakpoint of six (Cleveland and Liptzin 2007), indicating that the P availability tended to be constrained at high elevation. In addition, the average N/P value in mature leaves at low, middle and high elevation was 11.8, 13.5 and 18.4, respectively, again revealing the greater limitation of P relative to N at high elevation (Koerselman and Meuleman 1996; Reich and Oleksyn 2004). Therefore, we argue that the soil fertility in general decreased with increasing elevation in our study. This elevational change in soil fertility could be largely due to the decreasing temperature that could progressively inhibit the soil microbial activity and litter decomposition rate and thus make nutrients occluded in recalcitrant forms (Körner 2007; Sundqvist *et al.* 2011). We found that the decrease of soil fertility with increasing elevation was accompanied with the decrease of PASS (i.e. the greater resource conservation in both evergreen and deciduous species; Table 2). This is in agreement with the idea that plant species tend to have high nutrient conservation when they live in an infertile environment (Aerts 1999; Ordoñez *et al*. 2009; Freschet *et al.* 2010).

On the other hand, a significantly positive relationship between MAT and PASS in both evergreen and deciduous species was also found in our study (Fig. 4), consistent with the global pattern where the colder the climate is, the higher the conservation in leaf economics is (Wright *et al.* 2005*b*; van Ommen Kloeke *et al.* 2012). The observed increase of recourse conservation with decreasing elevation-induced MAT in both evergreen and deciduous species supports the hypothesis that the role of environmental filtering in community assembly increases with elevation (Callaway *et al.* 2002; Read *et al.* 2014). Specifically, under cold conditions at high elevation, the stronger conservation in leaf economics promotes leaf trait syndromes associated with superior stress tolerance but inferior competition, reflecting that the trade-off between resource competition and cold stress tolerance mediates the co-occurrence of evergreen and deciduous species at high elevation (Read *et al.* 2014). By contrast, in subtropical hot and humid environments at low elevation, warm temperature stimulates resource acquisition and promotes the higher carbon gain per year of evergreen species than deciduous species through the spreading of CC over several seasons; this may explain why evergreen species tend to be particularly dominant at low elevations in tropical and subtropical mountains (Chabot and Hicks 1982; van Ommen Kloeke *et al.* 2012).

In addition, we should be aware that the combined effects of temperature and soil can lead to the developmental and physiological constraints decoupling some leaf economic trait associations across elevations (Körner 1989, 2007). In our study, deciduous species decreased LLS but increased LMA and CC with the increase of elevation (Table 2), suggesting that LLS is decoupled from the rest of LES in deciduous species. The decoupling of LLS from the rest of LES has also been observed in temperate deciduous Viburnum species (Edwards *et al.* 2014), in communities dominated by herbaceous or deciduous woody species (Funk and Cornwell 2013) and even in deciduous shrubs and tress at the global scale (Wright *et al.* 2004). We consider three possible reasons for the decoupling of LLS from the rest of LES in deciduous species in our study. First, earlier leaf shedding in deciduous species could be a plastic response to the elevation-induced shorter length of growing season and lower nutrient availability (Givnish 2002; Pornon *et al.* 2011; van Ommen Kloeke *et al.* 2012). However, a thicker leaf with higher LMA and CC could be necessary for deciduous species to withstand physical stresses of climate and herbivory at high elevation (Coley *et al.* 1985; Matsuki and Koike 2006), resulting in the decoupling of LLS and the other LES traits. Second, the negative relationship between *A*_mass_ and LMA in deciduous species (Table 2) revealed the key role of LMA in the carbon budget. Moreover, the lack of response in *N*_mass_, together with an increase in LMA, would lead to higher leaf C/N and higher area-based nitrogen content that could increase the ability to conserve resources at high elevation, which is consistent with the predictions made by LES theory (Read *et al.* 2014). Under these conditions, leaves with higher LMA might not need longer LLS to repay themselves in deciduous species, leaving LLS to vary with the physical constraints by climate and herbivory (Funk and Cornwell 2013). Finally, LLS could be influenced by flowering phenology due to the more or less simultaneous development of leaves and inflorescences during the spring bug break (Edwards *et al.* 2014). We found the spring bug break tended to be later with increasing elevation in deciduous species (data not shown), which paralleled the decrease of LLS with increasing elevation. The close relationship between LLS and floral developmental thus suggests that the evolution of LLS has been influenced by other factors such as pollination, fruit maturation and dispersal, leaving LLS far from the leaf carbon balance sheet in deciduous species (Edwards *et al.* 2014).

### Phylogenetic variation in leaf economics

Consistent with the findings of Wright *et al.* (2004) and Shipley *et al.* (2006), we found close inter-specific correlations between leaf economic traits (Table 3). Let us take some fundamental trade-offs for examples. The observed negative relationship between *W*_m_ and LLS revealed that species with higher allocation to the liquid phase processes relative to structural tissue are at the expense of leaf longevity (Shipley *et al.* 2006). There was a negative relationship between *A*_mass_ and LLS in this study. The decline in *A*_mass_ with increasing LLS is likely as a result of the combination of a decrease in leaf N_mass_ and an increase in LMA, because high values of *A*_mass_ result in rapid leaf growth, shading older leaves and favouring rapid N re-translocation from the old leaves to the young ones, and early leaf demise in the end (Reich *et al.* 1992; Givnish 2002). The positive relationship between CC and LLS in our study suggested a trade-off between leaf CC and benefit over time (Shipley *et al.* 2006). Species with higher CC and LLS could invest more energy in leaf tissues, such as formation of higher leaf structural carbon and nutrients. Expensive and long-lived leaves might contribute to lower growth rates, but they could be less susceptible to herbivores. These inter-specific relationships suggest that there are genetic constraints limiting the independent evolution of leaf economic traits (Wright *et al.* 2004; Donovan *et al.* 2011). In other words, the genetic constraints could limit the LES evolution if the co-existing tree species lack the genetic variation necessary to produce fit trait combinations. It is noteworthy that intra-specific correlations between leaf economic traits are not necessarily consistent with the LES, which could be a result of the relatively smaller range of trait variation within than between species (Donovan *et al.* 2011). But, in a recent meta-analysis, Read *et al.* (2014) found the strengths of relationship between LMA and N_mass_ were equal or greater within species relative to the relationships among species along elevational gradients. The above inter-specific and intra-specific considerations support the conclusion that there is an underlying genetic basis to the trait combinations that we documented along the elevational gradient.

We found the λ values of leaf economic traits were significantly higher than 0 but significantly lower than 1 **[see Supporting Information—Table S2]**, suggesting that although there is phylogenetic signal in leaf economic traits, they have evolved according to a process other than pure Brownian motion (Revell *et al.* 2008; Kamilar and Cooper 2013). The phylogenetic signal in leaf economic traits indicates that closely related species tend to have similar trait values due to shared ancestry and this trait similarity decreases as the phylogenetic distance between species increases, reflecting the genetic constraints on trait evolution (Penuelas *et al.* 2010; Walls 2011; Kamilar and Cooper 2013). However, phylogenetic signal in leaf economic traits was considered to be low because of their intermediate values of λ (Kamilar and Cooper 2013). Our results are in accord with previous observations that some leaf economic traits (i.e. LLS, LMA, *A*_mass_, N_mass_, P_mass_ and PNUE) have weak phylogenetic signal (e.g. Penuelas *et al.* 2010; Fu *et al.* 2012). But, phylogenetic signal is low or high depending strongly on the evolutionary process (Revell *et al.* 2008; Ackerly 2009). We found that the OU model explained the evolution of leaf economic traits better than the Brownian motion model at species level (Table 1). The OU model of trait evolution was also observed in LMA at clade level in a large dataset of vascular plants (Flores *et al.* 2014). Under the OU model, faster phenotypic evolution of trait can lead to lower phylogenetic signal, as the position of a lineage in phenotypic space becomes increasingly influenced by the position of the optimum rather than by shared ancestry (Revell *et al.* 2008). Such a negative relationship between phylogenetic signal and evolutionary rate is likely for our sample as the higher values of λ presented the lower values of α across leaf economic traits (Fig. 2). The negative relationship between phylogenetic signal and evolutionary rate was also observed across traits of height, leaf size and seed size in six woody plant clades (Acer, Aesculus, Ceanothus, Arbutoideae, Hawaiian lobeliads and the silversword alliance) (Ackerly 2009). Note that the α values varied from 5.39 in LLS to 18.34 in PNUE, suggesting obvious variability of evolutionary rate among leaf economic traits (Table 1). We consider that the obvious differences in evolutionary rate among leaf economic traits could be important for the process of LES evolution. For instance, the faster evolutionary rates of N_mass_, P_mass_ and PNUE could help close relatives rapidly diversify to fill new environments where the quicker adjustments of the resource level (e.g. N_mass_ and P_mass_) and use efficiency (e.g. PNUE) should be preferential for plant fitness. Moreover, the faster evolutionary rates of N_mass_, P_mass_ and PNUE could also lead to quicker adjustments to the fit trait combinations.

A more direct evidence of the genetic constraints on the evolution of LES is from the relationship between PASS distance and phylogenetic distance. Consistent with our Prediction (iii), we found a significantly positive relationship between PASS distance and phylogenetic distance across all species pairs and only across intra-elevational pairs of evergreen vs. deciduous species (Fig. 5), suggesting that genetic divergence explains a significant proportion of inter-specific variation in leaf economics. The significant phylogenetic signal in PASS again revealed the effect of phylogenetic distance on PASS **[see Supporting Information—Table S2]**. The tight relationship between PASS distance and phylogenetic distance implies that if the pair of evergreen vs. deciduous species is closely related, PASS distance in the pair tends to be lower, which is similar to the finding that faster growth of deciduous species over evergreen species is not consistent when they share close relatedness (Antúnez *et al.* 2001). Our result therefore supports the competition-relatedness hypothesis that distantly related species compete less strongly than closely related species because distantly related species differ in many functional traits that they can use in different microhabitats and thus escape competition (Cahill *et al.* 2008). Note that the average PASS distance between evergreen and deciduous species increased with increasing elevation (Table 2). This means that the frequency of distantly related evergreen and deciduous pairs with wide spacing of leaf economic values increases with increasing elevation (Graham *et al.* 2014).

## Conclusions

Our results demonstrate that evergreen species showed a more conservative leaf strategy while deciduous species exhibited a more acquisitive leaf strategy at each elevation. As elevation increased, both evergreen and deciduous species tended to have greater resource conservation, which corresponded to the decreases of temperature and soil fertility. This convergent increase of resource conservation with increasing elevation suggests that the role of environmental filtering in community assembly increases with elevation. We found close inter-specific correlations between leaf economic traits, suggesting that there are strong genetic constraints limiting the independent evolution of leaf economic traits. We found that phylogenetic signal tended to decrease with increasing evolutionary rate across leaf economic traits, suggesting that the genetic constraints are important for the process of trait evolution. We found a significantly positive relationship between PASS distance and phylogenetic distance across species pairs and an increasing average PASS distance between evergreen and deciduous species with increasing elevation, implying that the frequency of closely related evergreen and deciduous pairs with narrow spacing of leaf economic values decrease with increasing elevation. Taken together, our results suggest that elevation acts as an environmental filter to both select the locally adapted evergreen and deciduous species with sufficient phylogenetic variation and regulate their distribution along the elevational gradient based on their coordinated spreading of phylogenetic divergence and leaf economic variation.

## Sources of Funding

This research was made through grants to K.B. from the National Natural Science Foundation (31100285), West Light Foundation of the Chinese Academy of Sciences in 2012, Guangxi Natural Science Foundation (2013GXNSFBA019079) and Guangxi Scientific and Technological Project (1355007-3).

## Contributions by the Authors

K.B. and X.W. designed the field experiment. K.B. constructed the phylogeny and analysed the data. K.B., C.H., X.W. and D.J. contributed to the writing.

## Conflict of Interest Statement

None declared.

## Supporting Information

The following additional information is available in the online version of this article –

**Table S1.** Species trait means, PASS and ITS (internal transcribed spacer 1, 5.8S ribosomal RNA, internal transcribed spacer 2) sequence accession number in GenBank.

**Table S2.** Results of the phylogenetic signal tests for leaf economic traits and PASS. Pagel's λ statistic was calculated on log_10_-transformed data. The *P*-values of λ different from zero (P0) and unity (P1) are shown.

**Table S3.** Principal component analyses of leaf economic trait data.

Additional Information
