# Supplementary material for: Leaf economics of evergreen and deciduous tree species along an elevational gradient in a subtropical mountain
Source: AoB Plants. 2015 Jun 6;7:plv064. doi: 10.1093/aobpla/plv064 (PMC4571104; doi:10.1093/aobpla/plv064)
Supplement: Additional Information [file supp_7_plv064_index.html]

Leaf economics of evergreen and deciduous tree species along an elevational gradient in a subtropical mountain — Leaf economics of evergreen and deciduous tree species along an elevational gradient in a subtropical mountain — Additional Information 

# Leaf economics of evergreen and deciduous tree species along an elevational gradient in a subtropical mountain

## Additional Information

Additional Information

- Additional Information - Doc file
