# Supplementary material for: Leaf economics of evergreen and deciduous tree species along an elevational gradient in a subtropical mountain
Source: AoB Plants. 2015 Jun 6;7:plv064. doi: 10.1093/aobpla/plv064 (PMC4571104; doi:10.1093/aobpla/plv064)
Supplement: Additional Information [file supp_plv064_plv064supp.doc]

**Supporting Information**

Table S1. Species trait means, primary axis species score (PASS) and ITS (internal transcribed spacer 1,5.8s ribosomal RNA, internal transcribed spacer 2) sequence accession number in GenBank.

| Species | Family | Altitude | Leaf habit | LLS | LMA | CC | *W_m_* | *N_mass_* | *P_mass_* | *A_mass_* | *R_mass_* | PNUE | PASS | Number |
| --- | --- | --- | --- | --- | --- | --- | --- | --- | --- | --- | --- | --- | --- | --- |
| *Castanopsis fargesii* | Fagaceae | Low | Evergreen | 18.0 | 120 | 1.71 | 1.11 | 19.5 | 1.78 | 108 | 10.0 | 79 | 0.03574 | AY040383 |
| *Lithocarpus henryi* | Fagaceae | Low | Evergreen | 16.3 | 110 | 1.53 | 1.21 | 20.5 | 1.55 | 94 | 8.6 | 72 | 0.07980 | EF057110 |
| *Rhododendron stamineum* | Ericaceae | Low | Evergreen | 14.2 | 145 | 1.81 | 1.25 | 18.5 | 1.85 | 90 | 8.1 | 62 | -0.16180 | AF393435 |
| *Machilus leptophylla* | Lauraceae | Low | Evergreen | 19.6 | 132 | 1.75 | 1.01 | 21.2 | 1.72 | 88 | 7.2 | 58 | -0.26800 | FJ755430 |
| *Acer fabric* | Sapindacea | Low | Evergreen | 12.5 | 104 | 1.48 | 1.31 | 25.1 | 1.98 | 100 | 11.3 | 54 | 0.39256 | AF241486 |
| *Castanopsis carlessi* | Fagaceae | Low | Evergreen | 21.3 | 127 | 1.66 | 1.06 | 20.7 | 1.92 | 71 | 5.9 | 48 | -0.34350 | AY040372 |
| *Acer oliverianum* | Sapindacea | Low | Deciduous | 10.3 | 58 | 1.42 | 1.48 | 25.4 | 2.06 | 188 | 11.1 | 105 | 1.05560 | AY605423 |
| *Photinia beauverdiana* | Styracacea | Low | Deciduous | 7.4 | 63 | 1.23 | 1.56 | 24.2 | 2.21 | 172 | 13.1 | 101 | 1.22239 | JQ392492 |
| *Liquidambar formosana* | Altingiaceae | Low | Deciduous | 9.4 | 72 | 1.31 | 1.44 | 23.9 | 2.02 | 151 | 15.4 | 89 | 0.99367 | GU576669 |
| *Alniphyllum fortunei* | Styracaceae | Low | Deciduous | 8.3 | 87 | 1.35 | 1.61 | 26.3 | 1.99 | 194 | 12.3 | 103 | 1.08159 | AF396437 |
| *Melliodendron xylocarpum* | Rosaceae | Low | Deciduous | 6.3 | 52 | 1.12 | 1.65 | 28.3 | 2.52 | 210 | 16.0 | 108 | 1.65303 | AF396444 |
| *Quercus glauca* | Fagaceae | Middle | Evergreen | 20.1 | 138 | 1.83 | 1.09 | 20.7 | 1.55 | 85 | 8.1 | 60 | -0.40550 | AY040458 |
| *Lithocarpus hancei* | Fagaceae | Middle | Evergreen | 17.9 | 144 | 1.77 | 1.16 | 22.4 | 1.68 | 88 | 8.6 | 52 | -0.22110 | AY040451 |
| *Illicium majus* | Schisandraceae | Middle | Evergreen | 27.6 | 173 | 2.03 | 0.90 | 18.8 | 1.33 | 62 | 6.1 | 45 | -0.81890 | AF163733 |
| *Adinandra bockiana* | Pentaphylacaceae | Middle | Evergreen | 23.6 | 161 | 1.93 | 1.01 | 19.9 | 1.80 | 71 | 6.8 | 49 | -0.52190 | HM061548 |
| *Photinia davidsoniae* | Styracacea | Middle | Evergreen | 16.3 | 127 | 1.65 | 1.21 | 23.6 | 1.73 | 95 | 9.8 | 56 | 0.05255 | FJ810005 |
| *Acer davidii* | Sapindacea | Middle | Deciduous | 5.1 | 69 | 1.36 | 1.48 | 24.4 | 1.94 | 120 | 10.2 | 69 | 0.85190 | HM008392 |
| *Fagus lucida* | Fagaceae | Middle | Deciduous | 5.9 | 52 | 1.25 | 1.53 | 25.7 | 1.78 | 234 | 15.2 | 127 | 1.43363 | AY040507 |
| *Liquidambar acalycina* | Altingiaceae | Middle | Deciduous | 7.9 | 59 | 1.53 | 1.39 | 24.6 | 1.89 | 179 | 12.2 | 102 | 0.97037 | GU576668 |
| *Fagus longipetiolata* | Fagaceae | Middle | Deciduous | 6.9 | 72 | 1.48 | 1.60 | 25.1 | 1.85 | 201 | 13.8 | 111 | 1.11255 | AY040511 |
| *Pterostyrax psilophyllus* | Styracaceae | Middle | Deciduous | 8.9 | 95 | 1.56 | 1.34 | 26.9 | 1.75 | 142 | 11.5 | 72 | 0.62023 | AF396447 |
| *Rhododendron haofui* | Ericaceae | High | Evergreen | 26.8 | 192 | 2.03 | 0.77 | 18.1 | 0.86 | 63 | 4.3 | 47 | -1.28470 | AY962552 |
| *Taxus mairei* | Taxaceae | High | Evergreen | 38.5 | 213 | 2.24 | 0.69 | 17.4 | 0.81 | 55 | 3.8 | 42 | -1.64030 | JX680621 |
| *Tsuga chinensis* | Pinaceae | High | Evergreen | 43.1 | 227 | 2.39 | 0.59 | 17.0 | 0.88 | 29 | 2.8 | 24 | -2.18450 | EF395482 |
| *Quercus myrsinaefolia* | Fagaceae | High | Evergreen | 30.5 | 173 | 1.93 | 0.95 | 15.1 | 1.12 | 57 | 6.6 | 51 | -1.02810 | AF098414 |
| *Cleyera japonica* | Pentaphylacaceae | High | Evergreen | 18.8 | 128 | 1.67 | 0.73 | 20.2 | 1.32 | 40 | 5.2 | 26 | -1.01360 | HM061546 |
| *Eurya loquaiana* | Pentaphylacaceae | High | Evergreen | 22.5 | 142 | 1.76 | 0.85 | 16.3 | 1.09 | 65 | 3.1 | 54 | -1.02590 | AY626870 |
| *Ternstroemia kwangtungensis* | Pentaphylacaceae | High | Evergreen | 25.5 | 186 | 1.86 | 0.90 | 14.4 | 0.90 | 60 | 4.6 | 57 | -1.17550 | HM061521 |
| *Nothotsuga longibracteata* | Pinaceae | High | Evergreen | 41.6 | 200 | 2.31 | 0.64 | 16.7 | 0.77 | 47 | 3.5 | 37 | -1.83070 | EF395455 |
| *Acer sinense* | Sapindacea | High | Deciduous | 5.8 | 79 | 1.56 | 1.18 | 32.4 | 1.44 | 154 | 12.5 | 67 | 0.73679 | HM352663 |
| *Stranvaesia davidiana* | Rosaceae | High | Deciduous | 5.6 | 87 | 1.72 | 1.21 | 23.8 | 1.41 | 94 | 10.1 | 54 | 0.24242 | JQ392487 |
| *Acer flabellatum* | Sapindacea | High | Deciduous | 4.2 | 82 | 1.41 | 1.32 | 33.8 | 1.57 | 167 | 11.1 | 69 | 0.95540 | HM352657 |
| *Sorbus caloneura* | Rosaceae | High | Deciduous | 6.2 | 126 | 1.78 | 1.09 | 22.1 | 1.37 | 118 | 8.3 | 76 | 0.09829 | FJ810008 |
| *Styrax odoratissimus* | Styracaceae | High | Deciduous | 4.9 | 98 | 1.64 | 1.25 | 28.0 | 1.60 | 103 | 7.1 | 53 | 0.33547 | AF327461 |

Trait means are averaged from five to eight individuals per species

Abbreviations for traits are as defined in Table 1

Table S2. Results of the phylogenetic signal tests for leaf economic traits and primary axis species score (PASS). Pagel's λ statistic was calculated on log_10_-transformed data. The *P*-values of λ different from zero (*P_0_*) and unity (*P_1_*) are shown.

| traits | λ | *P_0_* | *P_1_* |
| --- | --- | --- | --- |
| LLS | 0.93 | <0.001 | <0.001 |
| LMA | 0.89 | <0.001 | <0.001 |
| CC | 0.80 | <0.001 | <0.001 |
| *W_m_* | 0.88 | <0.001 | <0.001 |
| *N_mass_* | 0.91 | <0.001 | <0.001 |
| *P_mass_* | 0.72 | <0.001 | <0.001 |
| *A_mass_* | 0.93 | <0.001 | <0.001 |
| *R_mass_* | 0.91 | <0.001 | <0.001 |
| PNUE | 0.73 | 0.006 | <0.001 |
| PASS | 0.94 | <0.001 | <0.001 |

Abbreviations for traits are as defined in Table 1

Table S3. Principal component analyses of leaf economic trait data.

|  | All species | Species at low altitude | Species at middle altitude | Species at high altitude |
| --- | --- | --- | --- | --- |
| Variation explained by the primary component (%) | 83.4 | 85.0 | 84.5 | 83.2 |
| Leaf trait | Loadings |  |  |  |
| LLS | -0.89 | -0.97 | -0.96 | -0.97 |
| LMA | -0.95 | -0.95 | -0.97 | -0.96 |
| CC | -0.93 | -0.95 | -0.96 | -0.91 |
| *W_m_* | 0.97 | 0.95 | 0.97 | 0.96 |
| N_mass_ | 0.83 | 0.86 | 0.82 | 0.88 |
| P_mass_ | 0.86 | 0.86 | 0.70 | 0.91 |
| *A_mass_* | 0.96 | 0.96 | 0.97 | 0.94 |
| *R_mass_* | 0.95 | 0.91 | 0.96 | 0.93 |
| PNUE | 0.87 | 0.87 | 0.92 | 0.74 |

Abbreviations are as defined in Table 1
